# The Regulation of cGAS

**DOI:** 10.1007/s12250-018-0005-6

**Published:** 2018-03-15

**Authors:** Meiguang Xiong, Suyun Wang, Yan-Yi Wang, Yong Ran

**Affiliations:** Wuhan Institute of Virology, Chinese Academy of Science, Wuhan, 430071 China

**Keywords:** cGAS, DNA sensing, Regulation, Autoimmune

## Abstract

The cGAS-MITA pathway of cytosolic DNA sensing plays essential roles in immune response against pathogens that contain DNA or with DNA production in their life cycles. The cGAS-MITA pathway also detects leaked or aberrant accumulated self DNA in the cytoplasm under certain pathological conditions, such as virus induced cell death, DNA damage, mitochondria damage, gene mutations, which results in autoimmune diseases. Therefore, the cGAS-MITA pathway must be tightly controlled to ensure proper immune response against pathogens and to avoid autoimmune diseases. The regulation of cGAS-MITA pathway at MITA-level have been extensively explored and reviewed elsewhere, here we provide a summary and perspective on recent advances in understanding of the cGAS regulation.

## Introduction

The innate immune system is the first line of defense against incoming pathogens. Recognition of pathogen-associated molecular patterns (PAMPs) is essential for initiating innate immune response and subsequent adaptive immune response. Germ-line-encoded pattern recognition receptors (PRRs) detect PAMPs in extracellular, endosomal compartment and cytoplasm, leading to a series of signaling events which subsequently result in induction of type I interferons and proinflammatory cytokines. While many types of PRRs have been well demonstrated, such as TLRs (Toll-like receptors), CLRs (C-type lectin receptors), NLRs (NOD-like receptors) and RLRs (RIG-I-like receptors), the cytosolic DNA sensing pathway is gradually uncovered in recent years (Cai *et al*. 2014; Takeuchi and Akira 2010). There are several DNA sensors that have been identified to date, such as DAI, AIM2, IFI16, DDX41, RNA polymerase III and cGAS (Chiu *et al*. 2009; Hornung *et al*. 2009; Nie and Wang 2013; Orzalli and Knipe 2014; Parvatiyar *et al*. 2012; Takaoka *et al*. 2007; Unterholzner *et al*. 2010). Subsequent studies, especially gene knockout studies, have demonstrated that the cyclic GMP-AMP (cGAMP) synthase (cGAS) is a predominant and general sensor of cytosolic DNA no matter it is pathogenic or self DNA (Gao *et al*. 2015; Sun *et al*. 2013; Wu *et al*. 2013).

cGAS is expressed in a wide variety of tissues but prominently expressed in immune tissues. There are two adjacent IFN-sensitive response elements (ISREs) in the promoter of the *cGAS* gene, making it an ISG that can be induced by type I IFN-stimulation (Ma *et al*. 2015a). cGAS not only detects cytoplasmic DNA produced by a large variety of DNA-containing pathogens, such as DNA viruses and bacteria, but also detects DNA produced during reverse transcription by retrovirus (Gao *et al*. 2013; Hansen *et al*. 2014; Herzner *et al*. 2015; Mankan *et al*. 2014). More interestingly, cGAS also displays important functions in immune defense against some RNA viruses with no DNA intermediates in their life cycles, although the precise mechanism is still unclear (Aguirre and Fernandez-Sesma 2017; Aguirre *et al*. 2017; Ma and Damania 2016; Schoggins *et al*. 2014). In addition, cGAS is essential for cellular senescence and antitumor immunity by sensing accumulated cytoplasmic DNA from DNA damage or tumor DNA taken up by phagocytes such as dendritic cells, respectively (Lau *et al*. 2015; Mackenzie *et al*. 2017; Wang *et al*. 2017a; Yang *et al*. 2017).

## The cGAS-MITA Signaling Pathway

Upon binding to DNA, cGAS dimerizes and forms a 2:2 complex with DNA, which induces a conformational change in the active site of cGAS. Activated cGAS catalyzes the synthesis of 2′3′-cGAMP from ATP and GTP (Kranzusch *et al*. 2014; Li *et al*. 2013; Sun *et al*. 2013). cGAMP then serves as a second messenger, which binds to the endoplasmic reticulum–resident adaptor protein MITA (also known as STING and ERIS (Wu *et al*. 2013; Zhang *et al*. 2013). Binding of cGAMP triggers conformational changes of the MITA homodimer and its subsequent translocation from the endoplasmic reticulum (ER), through the Golgi apparatus, to perinuclear microsomal compartments, accompanied with the recruitment and activation of kinases TBK1 and IKKα/β, which subsequently activate the transcription factors IRF3 and NF-κB respectively, ultimately leading to induction of type I interferons (IFNs) and proinflammatory cytokines (Cai *et al*. 2014; Ishikawa and Barber 2008; Saitoh *et al*. 2009; Zhong *et al*. 2008).

The cGAS-MITA pathway is critically essential in host immune defense against incoming pathogens, however, aberrant activation of the cGAS-MITA pathway can also lead to autoimmune and inflammatory disease, such as Aicardi Goutieres syndrome (AGS) and STING-associated vasculopathy with onset in infancy (SAVI) (Chen *et al*. 2016b; Gao *et al*. 2015; Liu *et al*. 2014; Pokatayev *et al*. 2016). Thus, the cGAS-MITA pathway must be tightly regulated to maintain immune homeostasis. Since the regulation of MITA is relatively clear and has been discussed extensively elsewhere (Chen *et al*. 2016b; Ma and Damania 2016; Ran *et al*. 2014), here we focus on the regulatory mechanisms of cGAS.

## Regulation of cGAS by Post-translational Modifications

Post-translational modifications (PTMs) including phosphorylation, ubiquitination, and glutamylation have been reported to play important roles in the regulation of the cGAS-MITA pathway. For examples, glutamylation of cGAS impairs its ability to bind to DNA (Xia *et al*. 2016), whereas phosphorylation and K63- or K27-linked polyubiquitination of MITA is important for its activation and recruitment of TBK1 and IRF3 (Ran *et al*. 2014; Tsuchida *et al*. 2010; Wang *et al*. 2014; Zhang *et al*. 2012).

### Regulation of cGAS by Phosphorylation

Akt, a critical protein kinase involved in cellular metabolism, cancer development and other cell process, was demonstrated to phosphorylate the S305 or S291 of human or mouse cGAS, respectively (Seo *et al*. 2015). These sites are in close proximity to the catalytic site of cGAS. Therefore, phosphorylation of cGAS on these sites inhibits the catalytic activity of cGAS, resulting in decreased cGAMP synthesis, which in turn impedes the induction of type I interferons upon DNA stimulation and HSV-1 infection. Akt-mediated the phosphorylation of cGAS acts as a brake to control antiviral response, whether there is a phosphatase to reverse the phosphorylation and turn on cGAS-mediated signaling is an interesting question to be explored.

### Regulation of cGAS by Ubiquitination

Ubiquitination is a general post-translational modification and plays essential roles in the regulation of innate immune signaling cascades (Bhoj and Chen 2009). Although it is well established that MITA is extensively regulated by ubiquitination, the role of ubiquitination on cGAS regulation is just starting to be characterized. The E3 ligase RNF185, expression of which is upregulated in Systemic Lupus Erythematosus (SLE) patients, has been shown to be involved in the regulation of cGAS activity (Wang *et al*. 2017b). RNF185 potentiates the activity of cGAS upon DNA virus infection by catalyzing the formation of K27-linked ubiquitination chains on K137/384 sites of cGAS, resulting in enhanced production of type I interferons. Knockdown of RNF185 or mutation of cGAS ubiquitination sites resulted in decreased production of cGAMP. However, how K27-linked ubiquitination of cGAS modulates its enzymatic activity is still unclear. Another study, which discussed in detail in the following text, showed that K48-linked ubiquitination of cGAS leads to its degradation in an autophagosome-dependent manner, although the E3 ubiquitin ligase responsible for this process is unknown (Chen *et al*. 2016a).

### Regulation of cGAS by SUMOylation

Similar with ubiquitination, SUMOylation is also a reversible post-translational modification and plays essential roles in modulating innate immune responses. Recently, two independent groups provide new insights into the roles of SUMOylation in cGAS regulation. Hu *et al*. (2016) showed that SUMOylation of cGAS promotes its stability. In detail, TRIM38, function as an E3 SUMO ligase, together with SENP2, a SUMO-specific protease, catalyzes dynamic SUMOylation of cGAS at K231 and K479. TRIM38 targets cGAS for SUMOylation in uninfected cells and during the early phase of viral infection, which prevents its K48-linked ubiquitination and degradation. In the late phase of infection, cGAS is deSUMOylated by SENP2 and subsequently degraded via proteasome. The SUMOylation- and ubiquitination-dependent dynamic regulation of cGAS stability ensures an efficient triggering of the cGAS-MITA pathway in the early phase of DNA virus infection, as well as its timely termination upon resolution of infection. Notably, in the same study, MITA is shown to be similarly regulated by SUMOylation.

In another study, Cui *et al*. (2017) showed that SENP7, also a SUMO-specific protease, positively regulates cGAS-mediated signaling. They found that cGAS is SUMOylated on K335, K372 and K382. Given that these SUMOylation sites are located in either DNA-binding surfaces or dimerization interface of cGAS, SUMOylation of cGAS at these sites impairs both its DNA-binding ability and its self-association. SENP7 interacts dynamically with cGAS upon HSV-1 infection and deSUMOylates cGAS, thus turns on the activation of cGAS. Notably, there are some discrepancies between these two studies, such as distinct SUMOylation patterns and different roles of SUMOylation in cGAS-mediated cytosolic DNA sensing pathway. Probably, these discrepancies are caused by different moiety of SUMO. Hu *et al*. focused on E3 ligase TRIM38-mediated SUMO-1 modification of cGAS, whereas Cui *et al*. mainly focused on SUMO2/3 modification of cGAS without specific E3 ligases. It is not surprising that different SUMO modifications have different effects on cGAS. Further studies are necessary to clarify these confusions.

### Regulation of cGAS by Glutamylation

Glutamylation is a post-translational modification which is catalyzed by tubulin tyrosine ligase-like (TTLLs) glutamylases and reversed by enzymes of the cytosolic carboxypeptidase (CCPs) family (Rogowski *et al*. 2010). Recently, Xia *et al*. (2016) found that cGAS is tightly regulated by glutamylation. Mice deficient in either CCP5 or CCP6 are susceptible to the infection of DNA viruses, as a result of the failed induction of type I interferons. They found that polyglutamylation mediated by TTLL6 at glu272 dampens the DNA binding activity of cGAS, whereas monoglutamylation mediated by TTLL4 at glu302 blocks the enzymatic activity of cGAS. On the contrary, CCP6 and CCP5, the counterparts of TTLL6 and TTLL4, mediate the deglutamylation of cGAS at glu272 and glu302, respectively, thus release the inhibitory effects of glutamylation. Through dynamic regulation of the glutamylation state of cGAS, TTLL4, TTLL6, CCP5 and CCP6 work cooperatively to modulate cGAS-mediated immune responses. However, how these enzymes are triggered or regulated to control the glutamylation and deglutamylation of cGAS is unclear. Further studies are needed to resolve this question and provide more insights for the understanding of cGAS regulation.

## Regulation of cGAS by Crosstalk with Other Pathway

### Crosstalk with Autophagy

It has also been shown that Beclin-1, an autophagy protein, negatively regulates cGAS-mediated innate immune responses (Liang *et al*. 2014). On one hand, Beclin-1 directly interacts with the NTase domain of cGAS in a DNA binding dependent manner through its CCD domain, which impairs the enzymatic activity of cGAS, resulting in decreased cGAMP synthesis and subsequent impairment of type I interferon induction by DNA virus infection. On the other hand, knockdown of cGAS decreases the formation of autophagosome upon DNA stimulation, which suggests that cGAS may be involved in the formation of autophagosome. By this way, beclin-1 enhances autophagy-mediated degradation of cytosolic pathogen DNA to prevent excessive cGAS activation. These findings showed that autophagy adopts two strategies, elimination of pathogen DNA or inhibition of cGAS enzymatic activity, to negatively regulate DNA sensing pathway to avoid excessive activation of immune response.

Interestingly, a recent study by Chen *et al*. (2016a) provides another piece of evidence for negative regulation of DNA sensing pathway by autophagy. They found that E3 ligase TRIM14 could stabilize cGAS by inhibiting the autophagic degradation of cGAS. The K48-linked ubiquitination of cGAS at K414 is a signal for p62, a cargo receptor of selective autophagy. Without virus infection, cGAS undergoes K48-linked ubiquitination at K414, leading to p62-dependent selective autophagic degradation of cGAS. Upon DNA virus infection, TRIM14, as an interferon-stimulated gene, is induced and recruits the deubiquitinating enzyme USP14 to cleave K48-linked ubiquitin chains of cGAS, thus inhibiting p62-cGAS interaction as well as the degradation of cGAS. These studies reveal a positive feedback regulation of cGAS-mediated signaling by TRIM14 and provide insights into the crosstalk between autophagy and type I IFN signaling in innate immunity.

### Crosstalk with Inflammasome

Viral infection triggers the production of type I interferons and activation of inflammasomes. There are many examples of crosstalks between type I interferons production signaling and inflammasome activation to balance these two processes (Guarda *et al*. 2011; Zhang *et al*. 2014). Wang *et al*. (2017c) found that cGAS is inhibited by caspases, essential components of inflammasome. Upon binding to DNA, AIM2 forms a complex with ASC and caspase-1, leading to the activation of caspase-1 (Fernandes-Alnemri *et al*. 2009; Hornung *et al*. 2009). Activated caspase-1 directly binds to and cleaves cGAS at D140/157, resulting in reduced cGAMP production and type I IFN induction. Importantly, caspase-1 only cuts cGAS upon DNA virus infection but not RNA virus infection. Consequently, AIM2-, ASC-, and caspase-1-deficient mice display enhanced resistance to DNA virus infection. In addition, Caspase-4, 5, and 11 can cut cGAS in conditions of non-canonical inflammasome activation (Wang *et al*. 2017c). Their findings reveal a role for inflammasome in the regulation of cGAS-mediated induction of type I IFNs and provided insights into the crosstalk between DNA virus induced innate immune responses and inflammasome activation.

## Regulation of cGAS by Viral Proteins

Viruses have evolved elaborate mechanisms to antagonize the innate immune system. Plenty of studies have demonstrated that MITA, an essential adaptor downstream cGAS, is targeted by various viruses for immune evasion (Ding *et al*. 2013; Kalamvoki and Roizman 2014; Ma *et al*. 2015b; Wu *et al*. 2015). Recent studies supply several lines of evidence that cGAS is also antagonized by virus for immune evasion.

It is well established that Kaposi’s sarcoma-associated herpesvirus (KSHV) LANA localizes to the nucleus of infected cells. However, an isoform of KSHV LANA lacking the N terminal, generated by internal translation initiation, is localized to the cytoplasm. This cytoplasmic isoform of KSHV LANA was demonstrated to recruit and antagonize cGAS (Zhang *et al*. 2016). Overexpression of this isoform binds to cGAS and reduces the phosphorylation of TBK1 and IRF3, resulting in impaired production of type I IFNs and antiviral response. However, the precise mechanism has not been fully elucidated yet.

In addition to cytoplasmic isoform of LANA, ORF52, a tegument protein of KSHV, inhibits DNA sensing of cGAS (Wu *et al*. 2015). The enzymatic activity of cGAS is inhibited in the presence of ORF52 upon DNA stimulation or DNA virus infection. ORF52 can bind to both DNA and cGAS, and these abilities are important for cGAS inhibition. Intriguingly, its binding to cGAS is not dependent on its binding to DNA. ORF52 is a conserved protein in gammaherpes-viruses and its homologs in MHV68, RRV and EBV can also inhibit cGAS, suggesting that this is a general mechanism for immune evasion by gammaherpesviruses.

Dengue virus (DENV) is a single positive-stranded RNA virus of the family *Flaviviridae*, without DNA production in their life cycle. Nevertheless, it is well established that cGAS plays important roles in innate immune responses against DENV infection. It is proposed that cGAS detects mitochondrial DNA released during DENV infection (Aguirre *et al*. 2017; Ma and Damania 2016; West *et al*. 2015). A recent study showed that cGAS is degraded during DENV infection (Aguirre *et al*. 2017). DENV encodes a protease cofactor NS2B that promotes cGAS degradation in an autophagy–lysosome-dependent manner to avoid the detection of mitochondrial DNA, which results in the inhibition of type I interferon production during DENV infection. It is worthy to note that DENV can also evade the innate immune response through cleavage of MITA by its NS2B3 protease (Aguirre *et al*. 2012; Yu *et al*. 2012). Therefore, DENV targets both cGAS and MITA to minimize the host innate immune antiviral response.

## Conclusions and Outlook

**Fig. 1:**
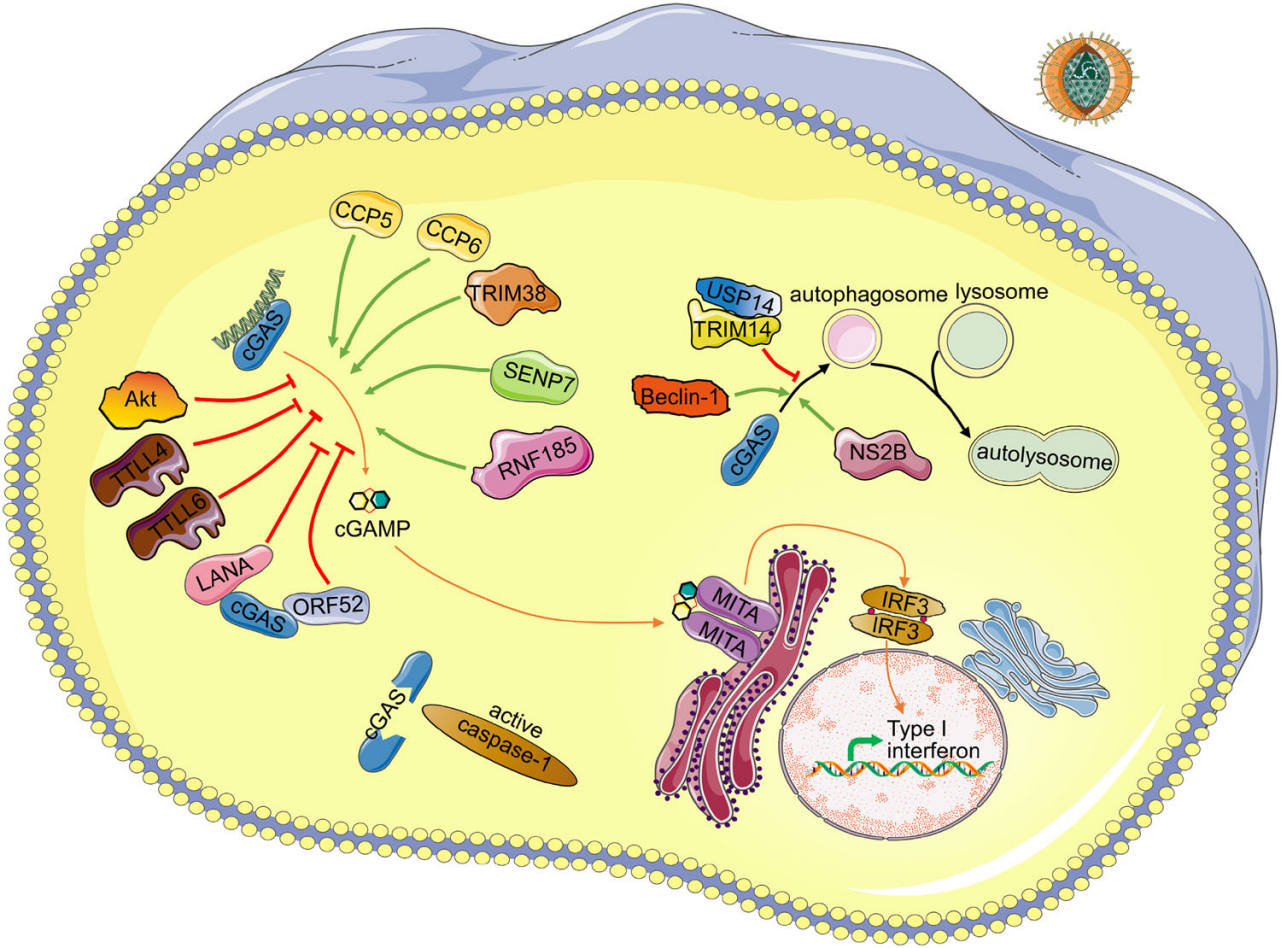
Schematic regulating network of cGAS. Detailed regulating proteins and virus antagonist of cGAS are depicted. Upon DNA binding to cGAS, cGAMP is synthesized by cGAS from ATP and GTP. Then binding of cGAMP to the adapter MITA induces conformational changes of MITA homodimer, leading to production of cytokines including type I interferons. Green arrows indicate positive regulators of the corresponding process. Red arrows indicate negative regulators of the corresponding process. Upon inflammasome activation, caspase-1 inhibits the cGAS-MITA pathway by cleavage of cGAS. DENV NS2B protein promotes the autophagy–lysosome-dependent degradation of cGAS. TRIM14 and USP14 inhibit the degradation of cGAS by cleaving K48-linked ubiquitin chains of cGAS. Beclin-1 promotes the autophagic degradation of cGAS. CCP5/6, TRIM38, SENP7 and RNF185 promote the activity of cGAS by deglutamylation, SUMOylation, deSUMOylation, ubiquitination of cGAS, respectively. TTLL4, TTLL6 and Akt inhibit the activation of cGAS by monoglutamylation, polyglutamylation, phosphorylation of cGAS, respectively. LANA of KSHV interacts and antagonizes cGAS. ORF52 of KSHV interacts with cGAS and inhibits the enzymatic activity of cGAS.

Since cGAS acts as an important cytosolic DNA sensor that leads to activation of innate immune antiviral responses, the activity of cGAS needs to be tightly modulated spatially and temporally by host factors to ensure the strength and duration of the immune responses. On the other hand, viruses have evolved elaborate mechanisms to antagonize the innate immune system. The cGAS-mediated signaling is targeted at every levels of signaling cascades by viruses. This review summarized the regulatory mechanisms of cGAS. The information presented in this review is summarized in Fig. 1 and Table 1. So far, our understanding of cGAS regulation is relatively limited. PQBP1 was reported to function as a coreceptor of cGAS in the sensing of retroviruses (Yoh *et al*. 2015), and studies also showed that IFI16 and cGAS work cooperatively during DNA virus infection in human foreskin fibroblasts (HFFs) and human keratinocytes (Almine *et al*. 2017; Orzalli *et al*. 2015). These studies indicate that the relationship between cGAS and other cytosolic DNA sensors needs to be further clarified, and also prompt us that there may be other coreceptor(s) or coactivator(s) of cGAS.

**Table 1:** Important regulation sites of cGAS.

Sites	PTMs	Regulators for PTMs	Proposed mechanism	References
K414	K48-Linked ubiquitination	E3 ligase unknown	Enhancing the degradation of cGAS by promoting cGAS-p62 interaction	Chen *et al*. (2016a)
	Deubiquitination	TRIM14-USP14	Deubiquitination of cGAS	
K173/384	K27-linked ubiquitination	RNF185	Potentiating the catalytic activity of cGAS	Wang *et al*. (2017b)
E302	Monoglutamylation	TTLL4	Inhibiting the synthase activity of cGAS	Xia *et al*. (2016)
	Deglutamylation	CCP5	Deglutamylation of cGAS	
E272	Polyglutamylation	TTLL6	Inhibiting the binding between cGAS and DNA	
	Deglutamylation	CCP6	Deglutamylation of cGAS	
K217/464	SUMOylation	TRIM38	Enhancing the stability of cGAS	Hu *et al*. (2016)
	DeSUMOylation	SENP2	DeSUMOylation of cGAS	
K335/372/382	SUMOylation	Unknown	Inhibiting the activity of cGAS	Cui *et al*. (2017)
	DeSUMOylation	SENP7	Potentiating the activity of cGAS	
S305	Phosphorylation	Akt	Suppressing the enzymatic activity of cGAS	Seo *et al*. (2015)
D140/D157	Cleavage	Caspase-1	Degradation of cGAS	Wang *et al*. (2017c)
Unknown	Cleavage	Caspase-4/5/11	Degradation of cGAS	Wang *et al*. (2017c)

cGAS is tightly regulated by post-translational modifications, such as phosphorylation, ubiquitination, SUMOylation, and glutamylation. However, there still are some questions to be answered. Firstly, it is demonstrated that cGAS undergoes ubiquitin-dependent degradation, but the E3 ubiquitin ligases responsible for this process is still unknown. Secondly, the roles of dynamic SUMOylation of cGAS need further clarification. Thirdly, whether other PTMs, such as acetylation, glycosylation, which are also responsible for the regulation of cGAS are worthy to explore. Most importantly, how these PTMs work cooperatively to modulate the initiation, duration, and termination of cGAS-mediated signaling is pivotal. Systemic study on these dynamic processes of PTMs *in vivo* during virus infection will provide more exciting and convincing insights into cGAS-mediated signaling.

Finally, the roles of cGAS in autoimmune diseases and antitumor immunity have drawn more and more attention, but the regulation mechanisms of cGAS in these processes are still largely unknown. Further investigation of these questions will certainly shed new lights on our understanding of cGAS-mediated DNA sensing pathways and help to develop strategies to prevent and treat the related diseases.

## Abbreviations

PAMP: Pathogen-associated molecular pattern
PRR: Pattern recognition receptor
TLR: Toll-like receptor
CLR: C-type lectin receptor
RLR: RIG-I-like receptor
ER: Endoplasmic reticulum
TBK1: TANK-binding kinase 1
IKK: IκB kinase
IRF3: Interferon regulatory factor 3
HSV-1: Herpes simplex virus 1
cGAMP: Cyclic GMP-AMP
cGAS: Cyclic GMP-AMP synthase
IFN: Interferon
ISG: Interferon-stimulated gene
ISRE: IFN-sensitive response element
AGS: Aicardi Goutieres syndrome
SAVI: STING-associated vasculopathy with onset in infancy
PTM: Post-translational modification
SLE: Systemic lupus erythematosus
TTLL: Tubulin tyrosine ligase-like
CCP: Cytosolic carboxypeptidase
KSHV: Kaposi’s sarcoma-associated herpesvirus
MHV68: Murine gammaherpesvirus-68
RRV: Ross River virus
EBV: Epstein–Barr virus
HFF: Human foreskin fibroblast
